# Glioblastoma metabolomics: uncovering biomarkers for diagnosis, prognosis and targeted therapy

**DOI:** 10.1186/s13046-025-03497-2

**Published:** 2025-08-07

**Authors:** Susan Costantini, Elena Di Gennaro, Giulia Fanelli, Palmina Bagnara, Chiara Argenziano, Carmen Maccanico, Marco G. Paggi, Alfredo Budillon, Claudia Abbruzzese

**Affiliations:** 1 Experimental Pharmacology Unit, Laboratori di Mercogliano, Istituto Nazionale Tumori-IRCCS-Fondazione G. Pascale, Napoli, 80131 Italy; 2 Cellular Networks and Molecular Therapeutic Targets, Proteomics Unit, IRCCS - Regina Elena National Cancer Institute, Rome, 00144 Italy; 3 Scientific Directorate, Istituto Nazionale Tumori-IRCCS-Fondazione G. Pascale, Napoli, 80131 Italy

**Keywords:** Glioblastoma, Metabolomics, Magnetic resonance, Mass spectrometry

## Abstract

Glioblastoma (GBM) is characterized by rapid growth, high molecular heterogeneity, and invasiveness. Specific aggressive factors are represented by MGMT promoter methylation, and IDH mutation status. Current standard-of-care for GBM includes surgical resection, followed by radiotherapy plus concomitant and adjuvant chemotherapy with temozolomide. However, patients almost invariably succumb due to therapy resistance and disease recurrences. Therefore, novel therapies for GBM are urgently needed to improve patient survival, necessitating the identification of new diagnostic and prognostic biomarkers, as well as therapeutic targets.

In this context, “omics” technologies, such as metabolomics and lipidomics, can generate vast amounts of data useful to elucidate the complex molecular mechanisms driving this disease, and discover potential novel biomarkers and therapeutic targets. Our review aims to highlight the current literature on the metabolomics studies conducted on GBM biological matrices, such as in vitro and in vivo models, tissues and biofluids, including plasma, saliva and cerebrospinal fluid.

From the data reported here, it appears that metabolic reprogramming in GBM is characterized by dysregulation in multiple pathways, particularly glycolysis (Warburg effect), amino acid metabolism, and the urea cycle, and the metabolic changes disclose promising tumor targets.

## Background

High-grade gliomas, particularly glioblastoma (GBM), represent the most prevalent and aggressive primary malignant brain tumors in adults, accounting for 24% of all central nervous system (CNS) cancers [1]. GBM is characterized by rapid growth, morphological and molecular heterogeneity, infiltration into surrounding healthy brain tissue, and resistance to current therapies.

The current standard-of-care GBM therapeutic protocol, regardless the status of the molecular indicators of disease severity, includes the maximal safe surgical resection while preserving neurological function, followed by radiotherapy plus concomitant and adjuvant chemotherapy with temozolomide (TMZ) [2, 3]. The efficacy of this chemotherapeutic protocol depends on the methylation status of the *O6-methylguanine-DNA methyltransferase* (*MGMT*) gene promoter, where high methylation levels are associated with increased tumor cell sensitivity to radio- and chemo-induced mutagenesis [4]. Therefore, testing for *MGMT* gene promoter methylation status is crucial for predicting the GBM response to therapy, where higher methylation levels correspond to a better patient outcome [5]. Median OS reaches 23.4 months in MGMT methylated GBM [6], and 66.8 months in the presence of both *MGMT* methylated and *isocitrate dehydrogenase (IDH)1* gene mutation [7].

Alternative therapies, including anti-Vascular Endothelial Growth Factor (VEGF) agents, e.g., bevacizumab, and brain-penetrating alkylating agents, e.g., lomustine (CCNU) and carmustine (BCNU), are employed as second-line treatments for recurrent GBM only, and provide marginal survival benefits [8, 9].

While GBM is typically characterized by an immunosuppressive tumor microenvironment and a low mutational burden (“cold” tumor), recent studies highlight the potential of immune checkpoint inhibitor (ICI) therapies in treating recurrent disease. Specifically, a combination of anti- Programmed cell death protein (PD)-1 and anti-Cytotoxic T-Lymphocyte Antigen (CTLA)-4 monoclonal antibodies has yielded a response rate of 12.46% [10].

Despite extensive research and the introduction of novel therapeutic strategies, GBM continues to pose a significant clinical challenge with limited treatment options. Patients almost invariably succumb due to therapy resistance and disease recurrences, leading to a grim median OS rate of approximately 6.2 months for those with recurrent disease [11, 12]. The primary drivers of GBM resistance and relapse, are glioma stem cells (GSCs), cancer subclones inherently resistant to radiation and chemotherapy [11, 13, 14]. In addition, cellular heterogeneity, tumor plasticity, and an immunosuppressive tumor microenvironment contribute to rapid increase in aggressiveness and relapse frequency [15, 16].

Post-treatment brain Magnetic Resonance Imaging (MRI) scans are routinely performed to assess GBM prognosis. This technique is highly sensitive in identifying cerebral lesions, but faces challenges in distinguishing tumor progression from pseudoprogression, a morphological trait representing post-radiotherapy sequelae and that may resolve spontaneously [17]. Recent studies demonstrate that patients with elevated gene expressions of *p53*, *X-ray repair cross-complementing 1* (*XRCC1*) and *interferon regulatory factor 9* (*IRF9*) [18] or methylation of the *MGMT* gene promoter [19] are associated with pseudoprogression. However, few validated biomarkers and/or clinical features currently exist for a definitive differentiation between progression and pseudoprogression. Brain tumor - reporting and data system (BT-RADS) score (https://btrads.com/), which some US institutions use, is validated by scientific studies and often help in the determination between progression and pseudoprogression [20]. Other institutions use neurologic assessment in neuro-oncology (NANO) criteria not only in research, but also in clinical practice [21].

Overall, we can underline that it is compulsory to identify and validate new approaches for swiftly predicting GBM progression, pseudoprogression, patient outcome, and treatment response. In this context, liquid biopsy is emerging as a useful tool to detect and quantify tumor-derived substances released into various body fluids, as urine, saliva, blood and cerebrospinal fluid (CSF). This technique involves quantifying circulating DNA, circulating tumor cells, extracellular vesicles, proteins, microRNA (miRNA)s and metabolites capable of crossing the blood–brain barrier (BBB) [22, 23].

Omics sciences, encompassing genomics, epigenomics, transcriptomics, proteomics, and metabolomics, generate extensive datasets, offering a powerful approach to elucidate the complex molecular mechanisms driving this disease. This knowledge is instrumental in developing personalized treatment strategies in precision medicine for GBM treatment [24–26].

Presently, omics profiling identifies three GBM subtypes: proneural, classic, and mesenchymal [27], each characterized by different genetic alterations and molecular signatures, as *epidermal growth factor receptor* (*EGFR*) gene amplification or mutation, *telomerase reverse transcriptase* (*TERT*) promoter mutation, homozygous loss of *cyclin-dependent kinase inhibitor 2 A/2B* (*CDKN2A/2B*) gene, *phosphatase and tensin homolog* (*PTEN*) gene deletion or mutation, gain of chromosome 7, and loss of the entire chromosome 10 [27, 28].

Specifically, metabolomics stands out as a powerful tool for identifying and quantifying a wide range of metabolites found in cells, tissues, or biological fluids. Through metabolome profiling, we can gain insights into the metabolic alterations associated with cancer, thus enabling the development of personalized treatment strategies tailored on each patient’s unique metabolic profile. Therefore, focusing on circulating metabolomic and lipidomic profiles allows for the identification of dynamic changes in key metabolites, including lipids, nucleotides, carbohydrates, and amino acids, that influence cellular signaling pathways involved in tumor progression and treatment resistance [29].

Metabolomics studies can be conducted using different methodologies, among which nuclear magnetic resonance (NMR), magnetic resonance spectroscopy imaging (MRSI), and mass spectrometry (MS) coupled to liquid chromatography (LC) or gas chromatography (GC).

NMR is a relatively fast methodology, with an acquisition time of a few minutes per sample, thus allowing the analysis of many samples per day, especially in automated platforms. Due to the overlap of metabolite signals, the analytical method suffers from low sensitivity and limited resolution [30]. High resolution magic angle spinning (HRMAS) NMR approach enables the evaluation of metabolite profiles in intact tissues [31, 32].

MRSI is a non-invasive imaging technology that generates detailed three-dimensional (3D) anatomical images. MS is preceded by LC or GC column-based separation techniques to minimize ion suppression effects, which can hinder the detection of less abundant metabolites. High-performance LC (HPLC) offers superior selectivity and separation efficiency compared to other chromatography methods. Separation degree, sensitivity and peak capacity have been greatly improved with the advent of ultra-performance liquid chromatography (UPLC) [33, 34]​​.

**Fig. 1:**
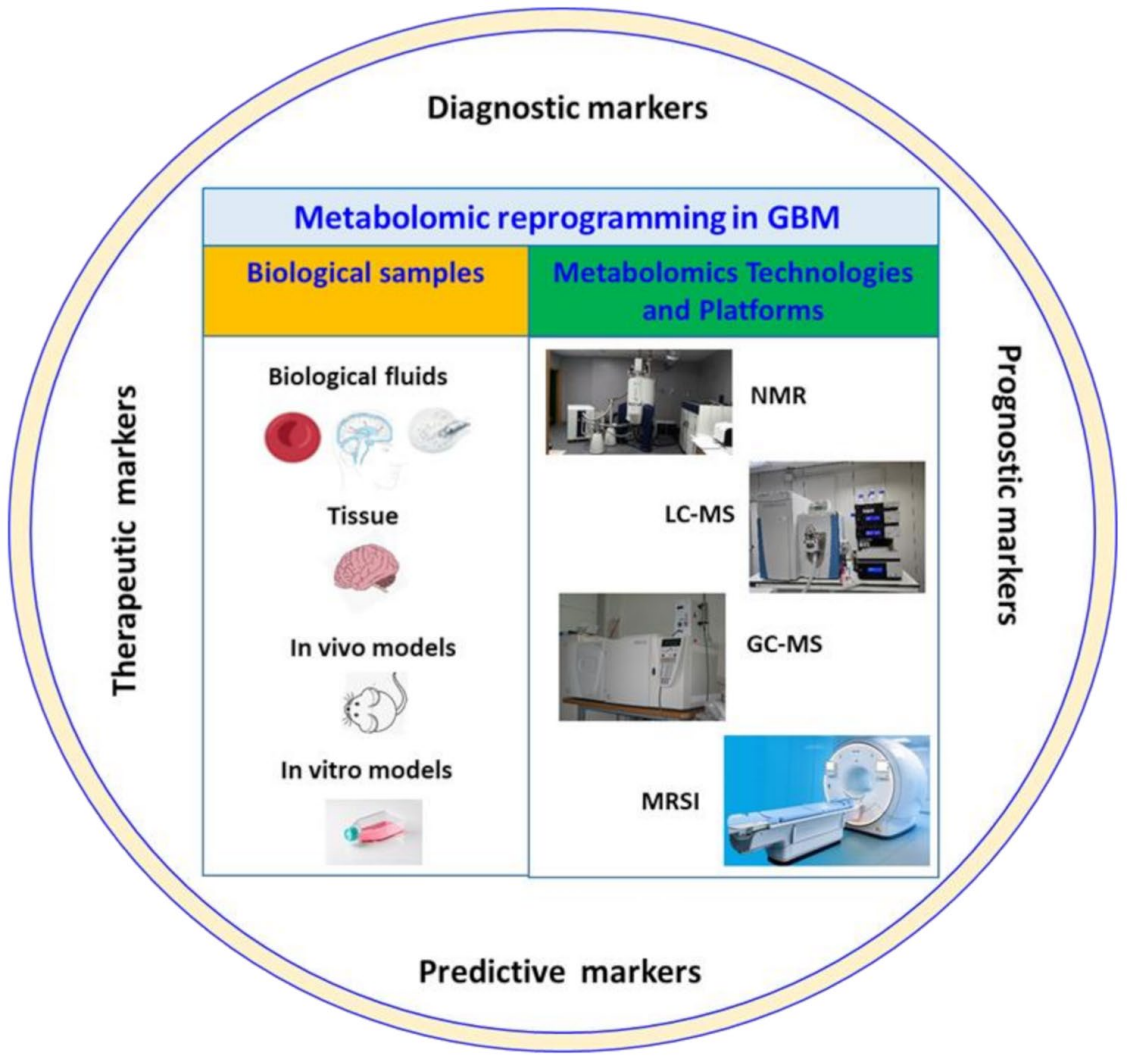
Schematic representation of malignant glioma and GBM biological matrices, i.e., in vitro and in vivo models, tissues and biofluids, on which different metabolomics approaches (NMR, LC-MS, GC-MS and MRSI) are applied to identify novel potential diagnostic, prognostic, predictive and therapeutic markers

This review aims to provide an overview of the current literature on the metabolomics studies conducted on GBM biological matrices, such as in vitro and in vivo models, tissues and biofluids, through which potential metabolites capable of predicting GBM progression [35, 36] and survival outcome [29] have been identified (Fig. 1).

## Metabolomics studies on GBM in vitro models

In the last years, various studies have been conducted on established and primary GBM cell lines, grown either in two-dimensional (2D) or 3D fashion, with the aim to highlight the key factors involved in metabolomics reprogramming correlated to malignant gliomas and GBM, also considering the presence of *IDH* gene mutation, the *MGMT* gene methylation status or the response to different treatments (Table 1).

**Table 1:** Summary of the metabolomics studies on GBM in vitro **models**

Comparisons	Metabolite levels	Technique	References
**Malignant cells** ***versus*** **Normal cells**	↑ Fumarate, G6P, glutamate, and succinate; ↓ Citrate	GC-MS	[36]
	↑ Tryptophan, methionine, kynurenine, and 5-methylthioadenosine	LC-MS	[39]
**Higher malignancy grade cells** ***versus*** **to lower malignancy grade cells**	↑ GSH	^1^H- NMR	[37]
**Genetic Profile**			
*PDGFRA + and EGFR-*	↑ Choline, and its derivatives (phosphocholine and glycerophosphocholine)	^1^H-NMR	[38]
*SLC38A1+*, *SLC7A8+*, *and SLC1A+*	↑ Aspartate, citrate, glutamine, and glutamate	^1^H-NMR	[38]
*Astrocyte elevated gene-1 (AEG-1) -knockout U251 cell line vs. U251 cell line*	↓ Choline/creatine, and lactate/creatine	^1^H-NMR	[40]
*IDH mutations*	↑ 2-HG; ↓Glutamate, GSH, lactate, and phosphocholine	^1^H-NMR	[41]
**Treated cell lines** ***versus*** **untreated**			
*Everolimus*	↓ Glutamate	LC-MS	[42]
*Plasma-activated medium (PAM)*	↑ Ribulose 5-phosphate, ribose-5-phosphate, xylulose 5-phosphate, and sedoheptulose 7-phosphate; ↓ Acetyl-CoA, malate, and fumarate	Capillary electrophoresis and MS	[43]
*Temozolomide-resistant*	↑ Myo-inositol, phosphatidylcholines, and phosphatidylethanolamines; ↓ GSH, polyunsaturated fatty acids, sphingomyelins, and taurine	MS	[44]
*Temozolomide and narginin*	↑ Palmitic acid, and sphingosine; ↓ Adenine, adenosine, acetyl-L-carnitine, choline, creatine, C8-Carnitine, DLCarnitine, L-Hexanoylcarnitine, propionylcarnitine, and spermine	MS	[45]
*Glucose*	↑ Creatine, uridine, and acetate	^1^H-NMR	[46]
*Lactate*	↑ Adenine, alanine, caprate, formate, succinate, uracil, and uridine	^1^H-NMR	[46]
*Glutamine*	↑ Adenine, glycolate, myo-inositol, and uracil	^1^H-NMR	[46]
*Glutamate*	↑ AMP, adenine, alanine, leucine, NADP+, pantothenate, phenylalanine, succinate, threonine, uracil, and valine	^1^H-NMR	[46]
*Ringer’s lactate solution irradiated by non-thermal plasma*	↑ acetyl CoA, alanine, cysteine, GSH, lactate, and pyruvate	Capillary electrophoresis and MS	[47]
*Polysaccharides from Cibotium barometz*	↓ Cysteine, glutamate, and glycine	^1^H-NMR	[48]
**Neurosphere** ***versus*** **monolayer**	↑ Glycine/myo-inositol, choline/creatine, and phosphocholine/creatine; ↓ Phosphocholine/glycerophosphocholine, and glycine/choline	HRMAS NMR	[49]
	↑ GSH/GSSG	^1^H- NMR	[37]
	↑ 5-methyl-THF, arginine, choline, guanine, guanosine, hypoxanthine, inosine, S-adenosylhomocysteine, and uracil; ↓ α-ketoglutarate, ATP, citrate, citrulline, creatine, creatinine, ornithine, taurine, uridine, valine, xanthosine, and xanthine	LC-MS	[51]
	Carnitine, glycerophospholipid, GSH, nucleotide, and tryptophan	LC-MS	[50]
**Organoids**			
*GSC versus non-GSC*	↓ lipid droplets		[55]
*LEGO versus wildtype*	↓glucose, glutamine, ATP, citric acid, aconitic acid, α-ketoglutarate, succinate, fumarate, and malate; ↑ cytidinediphosphocholine, DHAP, G3P, and lactate		[56]

Metabolomics evaluations on GBM cell lines show lower levels of citrate and higher of fumarate, glucose-6-phosphate (G6P), glutamate and succinate in malignant U-87 MG cells when compared with mesenchymal stem cells (MSC) [37], and higher levels of glutathione (GSH), a tripeptide that is composed of cysteine, glutamate, and glycine, in highly malignant cells (U-87 MG, U-118 MG and U-251 MG) when compared with less malignant ones (CHG5 and SHG44) [38]. Higher levels of choline, and its derivatives (phosphocholine and glycerophosphocholine) are observed in GBM cells characterized by high levels of *Platelet-Derived Growth Factor Receptor alpha* (*PDGFRA)* and low levels of *Epidermal Growth Factor Receptor* (*EGFR)* [39], whereas higher levels of aspartate, citrate, glutamine and glutamate correlate with the over-expression of genes for some transporters, such as *Solute Carrier Family 38 member 1* (*SLC38A1)*, *Solute Carrier Family 7 member 8* (*SLC7A8)*, and *Solute Carrier Family 1 Member 1* (*SLC1A*) [39].

Four differentially regulated metabolites (tryptophan, methionine, kynurenine, and 5-methylthioadenosine) are found in four established GBM cell lines (LN18, LN229, U-118 MG, and U-87 MG) when compared with normal human astrocytes [40].

Lower choline/creatine and lactate/creatine ratios in *astrocyte elevated gene-1* (*AEG-1*)-knockout U-251 MG cell line correlate to higher early apoptosis rates, higher percentage of cells blocked in the G2/M phase and lower invasion and migration abilities when compared to naïve U-251 MG cells [41].

Since mutations in *IDH* are mainly found in lower-grade gliomas and secondary GBM, some authors have evaluated if *IDH* mutations lead to changes in the cellular metabolome, thus demonstrating lower levels of glutamate, GSH, lactate and phosphocholine and higher levels of 2-hydroxyglutarate (2-HG), a metabolite that specifically builds up in tumors bearing *IDH* mutations [42].

Several studies have been conducted to evaluate how cellular metabolome changes following different treatments. Exposure to the selective mTORC1 inhibitor to four pediatric low-grade glioma cell lines result capable of inhibiting glutaminase, which in turn leads to a decrease in glutamate levels [43]. Plasma-activated medium (PAM) culture of U-251SP cells decreases tricarboxylic acid cycle (TCA) cycle intermediates (acetyl-CoA, malate, fumarate), inhibits glycolysis through Adenosine Triphosphate (ATP)-dependent glycerate 3-phosphate production, and upregulates the pentose phosphate pathway (PPP) by increasing ribulose 5-phosphate, ribose-5-phosphate, xylulose 5-phosphate, and sedoheptulose 7-phosphate levels [44]. TMZ-resistant GBM cells exhibit higher levels of myo-inositol, phosphatidylcholines and phosphatidylethanolamines, which can alter membrane composition or signaling pathways, whereas lower levels of GSH, polyunsaturated fatty acids, sphingomyelins and taurine may impair cell membrane functions and antioxidant homeostasis [45].

The combined treatment with TMZ and naringin, a bioflavonoid with anti-cancer and lipid-lowering effects, reduces the levels of adenine, adenosine, acetyl-L-carnitine, choline, creatine, C8-Carnitine, DL-Carnitine, L-Hexanoylcarnitine, propionylcarnitine, and spermine, while increasing palmitic acid and sphingosine [46].

Other authors have identified the following changes in culture media under specific conditions: (i) after glucose exposure, higher levels of creatine and uridine in U-251 MG cells and higher levels of acetate in U-87 MG cells; (ii) after lactate exposure, higher levels of adenine, alanine, caprate, formate, uracil, and uridine in U-251 MG cells and higher succinate levels in U-87 MG cells; (iii) after glutamine exposure, higher levels of adenine, glycolate, uracil, and myo-inositol in U-251 MG compared to U-87 MG cells; and (iv) after glutamate exposure, higher levels of Adenosine MonoPhosphate (AMP), adenine, alanine, Nicotinamide Adenine Dinucleotide (NADP)+, pantothenate, phenylalanine, threonine, and uracil in U-251 MG cells, and higher levels of leucine, succinate, and valine in U-87 MG cells [47].

Moreover, treatment of U-251SP cells with Ringer’s lactate solution, irradiated by non-thermal plasma, leads to increased levels of cysteine, GSH, pyruvate, lactate, acetyl CoA, and alanine [48]. On the other hand, treatment of U-87 MG cells with polysaccharides from *Cibotium barometz* decreases cysteine, glutamate and glycine, all factors involved in GSH biosynthesis, thus suggesting an inhibition in its synthesis and metabolism [49].

Another discussion point regards the cellular metabolic modifications induced in 2D or 3D culture conditions. In cells grown in neurosphere culture media, higher levels of glycine/myo-inositol, choline/creatine and phosphocholine/creatine ratios, and lower of phosphocholine/glycerophosphocholine and glycine/choline ratios are found, all indicators of high-grade, more malignant gliomas [50]. In three malignant glioma cell lines with different stemness characteristics, specific alterations at the level of carnitine, glycerophospholipid, GSH, nucleotide and tryptophan [51], as well as higher GSH/glutathione disulfide (GSSG) ratio [38], are associated with stem-like cell self-renewal and differentiation features. Metabolomics of U-87 MG and patient-derived NCH644 stem-like GBM cells, both exposed to neurosphere or monolayer culture conditions, highlight the following: (i) in U-87 MG growing in neurosphere conditions, higher levels of arginine, choline, guanine, guanosine, hypoxanthine, inosine, S-adenosylhomocysteine, and uracil, and lower levels of a-ketoglutarate, citrulline, taurine, uridine, xanthine are found, when compared to U-87 MG growing in monolayer; (ii) in NCH644 cells growing in neurosphere condition, higher levels of 5-methyl-THF and hypoxanthine, and lower levels of ATP, citrate, citrulline, creatine, creatinine, ornithine, valine, xanthosine and xanthine are detected when compared to NCH644 growing in monolayer; and, hence, (iii) four metabolic pathways (Alanine, aspartate and glutamate metabolism, Arginine biosynthesis, Citrate cycle and Pyrimidine metabolism) appear modulated in both cell lines grown in monolayer versus neurosphere culture medium [52].

Additionally, it is important to point out that organoids approximate human biology in a more comprehensive way than differentiated cell lines [53]. Several models of GBM organoids can be generated, each offering unique insights: (i) genetically engineered organoids, involving the genetic activation of oncogenes; (ii) patient-derived tumor organoids replicating the tumor microenvironment by including stromal vessels and immune cells; (iii) invasion models, using GSC to invade normal induced pluripotent stem cell (iPSC); (iv) GSC-derived organoids plus Epidermal Growth Factor/Fibroblast Growth Factor (EGF/FGF); and (v) patient-derived GSCs organoids developed without external growth factor supplementation [54]. Shakya et al. investigate gene expression in distinct microenvironments using patient-derived tumor organoids since cancer stem cells (CSCs) can alter lipid metabolism revealing significant differences in lipid processing gene expression and total lipid content among diverse cell populations from the same patient. They highlight a lower lipid droplet accumulation in CSCs compared to non-CSCs within these organoid models, proposing that lipid levels may not solely be a product of the microenvironment but could also reflect the cellular state [55]. In another study, metabolomic analysis of one-month-old iPSC-based human GBM-like organoid models engineered via loss of tumor suppressors through CRISPR/Cas9 (Clustered Regularly Interspaced Short Palindromic Repeats/CRISPR associated system) shows the activation of phospholipid synthesis and glycerol phosphate shuttle accompanied by the increased levels of dihydroxyacetone phosphate (DHAP), glycerol-3-phosphate (G3P), and cytidinediphosphocholine when compared to wildtype organoids. Moreover, these organoids exhibited higher levels of lactic acid, and lower levels of glucose, glutamine and TCA metabolites (ATP, citric acid, aconitic acid, α-ketoglutarate, succinate, fumarate and malate) [56].

Overall, these data indicate that the metabolomic reprogramming of GBM cells drives them towards the Warburg effect, epitomized in an increase in glycolysis and lactic acid production coupled with a decrease in citric acid cycle activity, as well as increase of PPP, GSH, glutamate, methionine and kynurenine metabolisms, urea cycle, and beta-oxidation of fatty acids. These metabolic shifts are directly linked to the need for enormous quantities of essential building blocks, such as amino acids, nucleotides and lipids, all required for rapid tumor growth.

## Metabolomics studies on GBM in vivo models

Given the notoriously limited survival time of GBM patients, identifying therapeutic approaches effective at the earliest stages of the disease is fundamental. For this reason, recent research efforts are increasingly focusing on new clinical strategies, including the evaluation of cerebral metabolites involved in GBM development and modulated during the treatment in in vivo models (Table 2).

**Table 2:** Summary of the metabolomics studies on GBM in vivo models

Models	Metabolite levels	Technique	References
**RCAS-PDGF mice**	↓ NAA; ↑ Lactate, and lipid signals *in GBM brain*	^1^H-NMR	[57–58]
**C57BL/6 mice (injection of murine glioma GL261 cells)**	↑ Arachidonic acid, gluconate 6-phosphate, G6P, glycine, fructose 6-phosphate, linoleic acid, oleic acid, palmitic acid, phosphatidylinositol, ribose 6-phosphate, UDP glucose, and UDP N-acetyl glucosamine	MALDI-MSI	[59]
**Advanced-stage C6 glioma rat model (stereotactic injection of C6 cells)**	↑ Alanine, lactate and taurine, sum of choline, glycerophosphocholine and phosphocholine, and myo-inositol *in GBM group versus sham group*	HRMAS NMR	[60]
**GL261 and LN229 tumor-bearing mice**	↓ Phospholipids and tryptophan; ↑ Saturated and unsatured fatty acids, proline, and nucleic acids	RAMAN SPECTROSCOPY	[61]
**Glioma xenograft harboring** ***IDH*** **mutation**	↓ Phosphoethanolamine; ↑ Glycerophosphocholine	(31)P-NMR	[62]
**Rat glioma models + TMZ treatment**	*In plasma*: ↑ Adenine, cis-9-10-epoxystearic acid, citraconic acid, citrate, D-mannose, D-glucose, L-allothreonine, and trans-4-hydroxy-L-proline; ↓ Aminobutyric acid, dimethylglycine, N-acetylneuraminic acid, pyridoxal phosphate, sarcosine, and xanthurenic acid	LC-MS	[63]
	*In brain*: ↑ O-acetyl-L-serine, L-serine, xanthosine, L-glutamate, sarcosine, N2-acetyl- L- ornithine, adenosine, anthranilic acid, and trigonelline		
**Isogenic GL261 GBM mouse model**	↓ Alanine, glutamate, glutamine, glycine, and lactate; ↑ Polyunsaturated fatty acids	MRS	[64]
**Orthotopic rat glioma model**	↓ Alanine and lactate; ↑ Creatine, inositol, and NAA *in GBM versus normal brain tissues*	^1^H-NMR	[65]
**Glioblastoma intracranial implantation mouse model (ketogenic diet and/or bevacizumab)**	↓ Aspartic acid and glutamic acid* in normal brain versus tumor after ketogenic diet plus bevacizumab*	GC-MS	[66]

Metabolic profiles of normal brain tissue and GBM were evaluated using a genetically engineered RCASTVA-J12p16/M9Pten mouse model generated by intracranial injection of cultured RCAS producing DF-1 cells expressing *PDGF* or *Kirsten rat sarcoma virus* (*Kras)* genes known to induce malignant cell transformation [57]. This study demonstrates that, in GBM-bearing brain of *RCAS-PDGF* mice, lower levels of N-acetylaspartate and higher levels of lactate and lipid signals at 1.3 ppm are detectable confirming the presence of intra- or extracellular lipid droplets, consistent with findings in other brain tumor models [58, 59]. Moreover, metabolomes in tumor, and healthy brain regions of C57BL/6 mice that received injection of murine glioma GL261 cells were compared, highlighting, in the tumor regions, higher levels of: (i) G6P and fructose 6-phosphate, indicative of increased glucose uptake to fuel glycolysis; (ii) glycine, derived from the conversion of G6P into 3-phosphoglycerate, which can then be converted in two steps to glycine; (iii) ribose 6-phosphate and gluconate 6-phosphate, both involved in the PPP pathway; (iv) Uridine diphosphate (UDP) glucose and UDP N-acetyl glucosamine, involved in hexosamine pathway; and (v) palmitic acid, linoleic acid, oleic acid, arachidonic acid and phosphatidylinositol [60].

Additionally, the evaluation of the metabolomic profile of tissues from an advanced-stage C6 glioma rat model, where Wistar rats underwent a stereotactic injection of C6 cells or cell medium (sham group), shows higher levels of alanine, lactate, taurine and the sum of choline, glycerophosphocholine and phosphocholine in GBM tumor compared to contralateral regions or sham rats that, in their turn, have similar levels of these metabolites. Of note, myo-inositol appears also elevated in both tumor and contralateral regions of GBM rats compared to sham rats [61]. More recently, a comparison between infiltration lesions and normal brain tissues in GL261 and LN229 tumor-bearing mice identified lower levels of phospholipids and tryptophan in the infiltrative lesions, whereas saturated and unsaturated fatty acids, amino acids, and nucleosides appear significantly higher, thus reflecting the enhanced mitotic rate of cancer cells. These metabolites are suggested to be crucial for GBM growth and thus potentially predictive of their infiltrative capabilities [62].

In addition, altered phospholipid homeostasis is implicated in *IDH*-mutated gliomas. Specifically, this study reveals decreased levels of phosphoethanolamine and increased levels of glycerophosphocholine in glioma xenografts harboring the *IDH* mutation. These findings are further corroborated in U-251 MG glioma cell line expressing the IDH-R132H protein mutation [63].

The metabolomics profile on murine models has also been evaluated to understand how the metabolome adapts to different treatments. Li et al. report that, in rat glioma models, TMZ treatment induces: (i) increased plasma levels of adenine, cis-9-10-epoxystearic acid, citraconic acid, citrate, D-mannose, D-glucose, L-allothreonine, and trans-4-hydroxy-L-proline, and decreased levels of aminobutyric acid, dimethylglycine, N-acetylneuraminic acid, pyridoxal phosphate, sarcosine, and xanthurenic acid; and (ii) in the brain increased levels of O-acetyl-L-serine, L-serine, xanthosine, L-glutamate, sarcosine, N2-acetyl-L-ornithine, adenosine, anthranilic acid and trigonelline [64]. From these data it can be argued that TMZ modulates metabolites involved in several pathways, including adenosine, sarcosine and adenosine deaminase [64]. In parallel, other authors characterize macrophage populations in an isogenic GL261 GBM mouse model, highlighting higher levels of polyunsaturated fatty acids and lower levels of alanine, glutamate, glutamine, glycine and lactate in TMZ responding mice compared to unresponsive ones [65].

In an orthotopic rat glioma model, treatment with vorinostat, a histone deacetylase inhibitor, for three days led to lower levels of alanine and lactate and higher levels of creatine, inositol and N-acetylaspartate bringing these metabolites closer to levels found in normal brain tissue [66].

A metabolome analysis of tissues from GBM intracranial implantation mouse models, treated with ketogenic diet and/or the VEGF inhibitor bevacizumab, demonstrates a decrease in aspartic and glutamic acid levels in tumors under ketogenic diet in combination with bevacizumab [67].

Overall, these in vivo studies highlight key metabolites involved in GBM onset and progression, confirming that metabolomic reprogramming in GBM is mainly based upon the Warburg effect, in addition to the modulation of the urea cycle and the metabolism of amino acids, GSH, and sphingolipids. Moreover, these data highlight that different treatments can significantly overmodulate the nucleotide and sarcosine pathways, and deregulate the amino acid metabolism, reflecting cancer cells’ altered amino acid consumption and processing to sustain their growth and proliferation.

## Metabolomics studies in GBM tissues

To gain a deeper understanding of the molecular mechanisms driving GBM evolution, recent research has increasingly focused on metabolomics investigations of GBM tissues to identify novel diagnostic biomarkers and therapeutic targets (Table 3).

**Table 3:** Summary of the metabolomics studies in GBM tissues

Comparisons	Metabolite levels	Technique	References
**GBM** ***versus*** **normal tissues**	↓ Phosphatidylcholine; ↑ L-palmitoylcarnitine, triacylglycerol, and stearoylcarnitine	LC-MS	[67]
	↑ Arachidonic acid, oleic acid, palmitic acid, phosphatidylethanolamine, and several phosphocholines; ↓ Specific fatty acids	Rapid evaporative ionization MS	[68]
**GBM** ***versus*** **oligodendrogliomas tissues**	↑ Mannitol, and phenylalanine; ↓ 4-aminobutyrate, creatinine, 2-HG, glycerol-2-phosphate, glycerol-3-phosphate, myo-inositol, and ribitol	GC-MS	[69]
	*In patients with longer survival*: ↑ Fructose, glycerol-3-phosphate, myo-inositol, and ribitol		
**Recurrent GBM tissues (rGB)** ***versus*** **initial GBM tissues**	↓ Ceramides, glycerolipids, sphingolipids, and triacylglycerols; ↑ Glycerophosphoglycerols	LC-MS	[70]
**Low grade** ***versus*** **grade IV**	↑ Myo-inositol/total choline *in low-grade* ↑ Phosphocholine/ glycerophosphocholine *as index of transformation to grade IV* ↑ 2-HG *in grade IV*	^1^H-NMR	[71]
	↑ Hypotaurine (correlated with GBM occurrence and grade)	Capillary electrophoresis and MS	[72]
***IDH*-mutated** ***versus*** **wildtype GB**	↑ 2-HG; ↓ Gamma-aminobutyric acid, and glutamate	MRSI	[73]
	↑ Choline, glycerophosphocholine, and phosphocholine *in grade II and III versus wildtype*		
	↑ 2-HG, AMP, ATP, creatine, myo-inositol, pyruvate, and scyllo-inositol; ↓ Fatty acyl chains, glutamate, sphingolipids, taurine, and triglycerides	MS	[74]
	↑ 2-HG, erythritol, glycerol-2-phosphate, and inositols; ↓ Sphingosine, and lysoglycerophospholipids	GC-MS and LC-MS	[75]
***IDH*-mutated GBM tissues** ***versus IDH*-mutated astrocytomas and oligodendrogliomas**	↓ NAA and aldopentose; ↑ isolecucine/leucine/valine, glycine, methionine, ornithine, proline, threonine, and tyrosine	GC-MS and LC-MS	[75]
**Cancer subpopulations within GBM regions**		Orbitrap secondary ion MS	[76]
*Necrotic cancer cells versus viable GBM cells*	↑ 8-hydroxy-7-methylguanine, cytosine, phosphate, purine, and xanthine		
*Viable tumor cells versus necrotic cancer cells*	↓ 4,6-dihydroxyquinoline, and serotonin		
*Necrotic and viable GBM cells versus non-cancerous cells*	↓ 5-hydroxytryptophol, 5-methoxytryptophol, 5-hydroxyindoleacetaldehyde, 5-methoxyindoleacetate, formyl-5-hydroxykynurenamine, indole-3-acetaldehyde, indole acetate, kynurenine, and tryptophan		
**Intratumoral heterogeneity within GBM**			
*In tumor core versus tumor edge*	↑ 4-hydroxy-phenylpyruvate	MS	[77]
*In edge samples versus core samples*	↑ Alanine, creatine, cystathionine, nicotinamide, and D-pantothenic acid; ↓ 2-oxobutyric acid, uric acid, threonine, and N1,N12-diacetylspermine	LC-MS	[78]
	↑ Itaconic, spermine and pantothenic acid; ↑ Hydroxyhexanoycarnitine *in presence of MGMT promoter methylation*		

Gilard et al. report differential expression of phospholipids, acylcarnitines, diacylglycerol, sphingomyelins, triacylglycerols, and steroid ester between GBM and normal tissues. In detail, GBM tissues show reduced levels of phosphatidylcholine and increased levels of L-palmitoylcarnitine, triacylglycerol and stearoylcarnitine compared to normal tissues [68].

Another study on GBM and control brain tissue samples reveals elevated levels of arachidonic acid, oleic acid, palmitic acid, phosphatidylethanolamine, and several phosphocholines, while concurrently demonstrating decreased levels of specific fatty acids in GBM [69].

Metabolite levels have been assessed in tumor samples from low-grade gliomas, high-grade gliomas, and patients with varying survival outcomes. Compared to oligodendrogliomas, GBM samples exhibit elevated levels of mannitol and phenylalanine, while displaying reduced levels of 4-aminobutyrate, creatinine, 2-HG, glycerol-2-phosphate, glycerol-3-phosphate, myo-inositol, and ribitol. Furthermore, GBM patients with longer OS exhibit significantly higher levels of fructose, glycerol-3-phosphate, myo-inositol, and ribitol. These last four metabolites are thus suggested as potential prognostic markers for favorable outcomes [70].

Comparative quantitative lipidomic analyses of patient-matched initial versus recurrent GBM (rGBM) reveal decreased levels of ceramides, glycerolipids, sphingolipids, and triacylglycerols, alongside increased levels of glycerophosphoglycerols. These findings highlight the critical role of ceramide regulation in GBM resistance. Indeed, chemo- and radiotherapy can stimulate sphingomyelinases, leading to the conversion of sphingomyelin into ceramide, whose subsequent accumulation can trigger apoptosis. Therefore, these findings underscore the critical role of ceramide regulation in mediating resistance to GBM treatment, especially in rGBM [71].

Given the significant intra- and inter-tumoral heterogeneity observed in GBM lesions, even within the same malignancy grade, a 2014 study investigated the metabolome of a large cohort of patients with newly diagnosed primary or recurrent gliomas (grades II-IV) using tissue samples. This research reveals the following key findings: (i) an increased ratio between myo-inositol and total choline characterizes recurrent low-grade gliomas; (ii) an elevated phosphocholine to glycerophosphocholine ratio indicates a potential transformation of the gliomas to grade IV; (iii) elevated 2-HG is associated to a grade IV lesion originating from a lower grade lesion [72].

Other authors highlight that higher levels of hypotaurine correlate strongly and positively with both GBM occurrence and grade, suggesting this oncometabolite as a potential indicator for GBM diagnosis and therapy [73].

Metabolic profiling of GBM tissues reveals significant metabolomic distinctions between *IDH*-mutated and wildtype GBMs, underscoring another crucial aspect of their metabolic diversity. Indeed, as expected, *IDH*-mutated tissues show higher levels of 2-HG and lower levels of gamma-aminobutyric acid and glutamate. Moreover, increased levels of free choline, glycerophosphocholine and phosphocholine are observed in grade II and III *IDH*-mutated samples [74]. In two studies on *IDH* mutant gliomas, the authors observe elevated levels of 2-HG, AMP, ATP, creatine, myo-inositol, pyruvate and scyllo-inositol as well as reduced levels of glutamate, taurine, and fatty acyl chains, sphingolipids and triglycerides, in turn correlate with lower protein expression levels of long-chain acyl-CoA synthetase 1 and 4, and very long-chain acyl-CoA synthetase 3 [75]. In addition, other authors identify higher expression of 2-HG, erythritol, glycerol-2-phosphate and inositols, and lower expression of sphingosine and lysoglycerophospholipids in GBM tissues bearing an IDH-mutation. Furthermore, when compared to *IDH*-mutated diffuse astrocytomas and oligodendrogliomas, *IDH*-mutated GBMs exhibit lower N-acetyl aspartate and aldopentose levels while displaying elevated levels of several amino acids (i.e., isoleucine/leucine/valine, glycine, methionine, ornithine, proline, threonine and tyrosine). With their work, these authors *de facto* identify unique metabolic profiles for the six most prevalent adult glioma subtypes. These findings hold significant potential to inform the development of non-invasive directed imaging techniques before surgery and guide the development of personalized therapeutic strategies [76].

Given the presence of diverse cancer subpopulations within GBM lesions, which often contribute to immune evasion and treatment failure, some researchers investigate the metabolism of distinct GBM regions, i.e., necrotic, viable tumor cells, and non-cancerous areas [77]. Necrotic cancer cells are distinguished from viable GBM cells based on their distinct metabolic profiles, characterized by elevated levels of 8-hydroxy-7-methylguanine, cytosine, phosphate, purine, and xanthine. On the other hand, viable tumor cells show decreased levels of 4,6-dihydroxyquinoline and serotonin compared to their necrotic counterparts. In both necrotic and viable GBM cells, the authors observe comparable levels of 5-hydroxytryptophol, 5-methoxytryptophol, 5-hydroxyindoleacetaldehyde, 5-methoxyindoleacetate, formyl-5-hydroxykynurenamine, indole-3-acetaldehyde, indole acetate, tryptophan, and kynurenine, while these levels are consistently lower than those found in non-cancer cells. All these findings underscore the crucial role of tryptophan metabolism in GBM cell survival [77].

Acknowledging the significant intratumoral heterogeneity of GBM, characterized by distinct core and infiltrating edge regions with unique microenvironments that drive tumor progression and invasiveness, other studies have investigated the metabolome in both these regions. It has been demonstrated that 66 out of 168 metabolites significantly differ between GBM paired regions of each patient. Top metabolic variations include: (i) higher expression of 4-hydroxy-phenylpyruvate, a downstream metabolite of tyrosine, within the tumor core versus the edge [78]; (ii) significantly elevated levels of alanine, creatine, cystathionine, nicotinamide, and D-pantothenic acid, while notably lower levels of 2-oxobutyric acid, uric acid, threonine, and N1,N12-diacetylspermine in edge versus core samples [79]; (iii) in cases with high *MGMT* promoter methylation status, elevated levels of hydroxyhexanoycarnitine and reduced levels of spermine in core samples, alongside higher levels of both itaconic and pantothenic acid are detected in edge samples [79]. The key metabolic differences identified between malignant glioma core and edge tissues collectively demonstrate the potential of machine learning to uncover novel prognostic and therapeutic targets.

Taken together, these data from tissue studies highlight metabolites implicated in molecular mechanisms driving GBM evolution, even in the presence of *IDH* mutation and *MGMT* promoter methylation, highlighting the strong involvement of lipid metabolism and polyamine, nucleotide, and amino acid-related pathways. Considerable evidence, as also shown in in vitro models, concerns the crucial role of 2-HG, an oncometabolite whose levels increase in the context of hypoxia or acidic pH, thereby influencing the immune cells fate.

## Metabolomics studies on GBM biological fluids

The advent of liquid biopsies has revolutionized cancer diagnostics by providing a non-invasive alternative to traditional tissue biopsies. Liquid biopsies, which typically involve the analysis of molecular components such as metabolites and lipids, enable differentiation between distinct tumor phenotypes and real-time monitoring of patients’ pathophysiological status. This approach provides critical insights into the temporal and spatial clonal evolution of tumors, representing a promising avenue for identifying novel biomarkers of cancer progression and therapeutic response [80, 81]. In recent years, various studies have been conducted across different biological fluids, e.g., plasma, saliva and CSF from GBM patients, even in the presence of *IDH* mutation or after treatments, to highlight metabolites involved in GBM evolution and progression that correlate with patient outcome, and predict therapeutic response (Table 4).

**Table 4:** Summary of the metabolomics studies on GBM biological fluids

Comparisons	Metabolites levels	Technique	References
**GBM** ***versus*** **other malignant** **glioma subtypes**	↑ Ornithine, and uridine (*plasma*)	LC-MS	[81]
	↓ 2-Oxoarginine, argininate, alpha-ketoglutarate, chenodeoxycholate, and cysteine (*plasma*)	LC-MS	[82]
	↑ Glucose, pyruvate, phenylalanine and tyrosine; ↓ Citrate, glutamine, and succinate (*plasma*)	^1^H-NMR	[83]
	↑ Citric and isocitric acids, 2-aminopimelic acid, and lactate (*CFS*)	GC-MS	[93]
**GBM patients** ***versus*** **controls**	↑ Cysteine, N-acetylglucosamine, creatinine, glycine, myo-inositol, and proline (*plasma*)	GC-MS	[84]
	Amino acid levels as prognostic biomarkers (*plasma*)	LC-MS	[85]
	↑ Putrescine, glutamate signaling and increased lipid metabolism (*CFS*)	MxP^®^ Quant 500 kit	[94]
	15 acylcarnitines, 14 amino acids, 10 biogenic amines, 1 cholestan steroid, 1 diacylglycerol, 75 phospholipids, 20 sphingomyelins and 12 triacylglycerids differently expressed (*plasma*)	LC-MS	[67]
**GBM patients harboring** ***IDH*** **wildtype**			
*Surgery*	↑ 2-hydroxy-5-sulfopyridine-3-carboxylic acid, 2-amino-3-methoxybenzoic acid, 3-cysteinylacetaminophen, acetaminophen sulfate, betonicine, dehydrofelodipinee, glycine, glycocholic acid, glycodeoxycholic acid, p-acetamidophenyl-beta-D-glucuronide, S-methyl-3-thioacetaminophen, and taurocholic acid; ↓ 1-methylnicotinamide, 1-hydroxymidazolam-beta-Dglucuronide, 3-hydroxybutryic acid, bupivacaine, dehydrofelodipine, hexadecanedioic acid, riluzole, lactitol, linoleic acid, mannitol, nudifloramide, and sorbitol (*plasma*)	LC-MS	[87]
*Post-Radiation versus Pre-Radiation*	↑ N-methylisoleucine, 4-methyl-5-thiazoleethanol, and 6-hydroxycaproic acid; ↓ 3-famotidine, N-isovalerylglycine, and methylcrotonylglycine (*plasma*)	LC-MS	[88]
*Post-Treatment versus Pre-Radiation*	↓ L-propionylcarnitine, 3-methylcrotonylglycine, N-isovalerylglycine, famotidine, 1,5-pentanediamine, chenodeoxycholic acid, 24-acyl-beta-D-glucuronide, acetaminophne sulfate, and 2-amino-3-methoxybenzoic acid; ↑ N-methylisoleucine, coniferylaldehyde, 4-methyl-5-thiazoleethanol, dimethylsulfoxide, glycerophosphocholine, diatrizoic acid, and bradykinin (*plasma*)	LC-MS	[88]
***IDH*** **mutation** ***versus IDH*** **wildtype**	↑ Arginine, N-acetylputrescine, nicotinate, glucosamine, methionine, and trimethylamine-N-oxide (*plasma*)	LC-MS	[81]
	Metabolic reprogramming of pyruvate (*plasma*)	MRS	[41]
	↑ Ethanolamine, glycerophosphocholine, and glycerophosphorylethanolamine, and phosphocholine (*plasma*)	MRS	[89]
	↑ Citric acid, isocitric acid, and lactate (*CSF*)	GC-MS	[93]
**Outcome and survival**			
*Poor outcome and survival*	↑ Kynurenate (*plasma*)	LC-MS	[89]
	↑ (±)-(Z)-2-(5-Tetradecenyl) cyclobutanone, and hippuric acid (*plasma*)	LC-MS	[90]
	↑ Pentose phosphate pathway and the Warburg effect; Greater heterogeneity in lipid abundance (*plasma* and *saliva*)	LC-MS	[28]
*Good outcome and survival*	↑ Arginine, and methionine (*plasma*)	LC-MS	[89]
	↑ 2,6-diisopropyl-3-methylphenol, 7-ketocholesterol, dopamine, perlolyrine, and piperidine (*plasma*)	LC-MS	[90]

Plasma metabolomic profiling reveals elevated levels of ornithine and uridine in GBM compared with lower grade malignant glioma patients [82]. Some metabolites (2-oxoarginine, argininate, alpha-ketoglutarate, chenodeoxycholate and cysteine) showed an inverse correlation with overall glioma risk. The same research group demonstrates the involvement of xanthine metabolites in glioma development and progression [83]. Baranovicová et al. compare the metabolome of GBM patients to those with low grade gliomas (astrocytoma, meningioma and oligodendroglioma) and healthy volunteers (controls). They find lower levels of citrate, glutamine and succinate, and higher levels of glucose and pyruvate in primary brain tumor patients relative to controls. On the other hand, higher levels of phenylalanine and tyrosine are evident in GBM patients only [84]. Jonsson et al. identify increased levels of cysteine, N-acetylglucosamine, creatinine, glycine, myo-inositol, and proline in glioma cases compared to controls, with these changes associated with glioma progression [85]. Other authors highlight 15 acylcarnitines, 14 amino acids, 10 biogenic amines, 1 cholestan steroid, 1 diacylglycerol, 75 phospholipids, 20 sphingomyelins and 12 triacylglycerids as differently expressed in the plasma of GBM patients compared to controls [68]. Bobeff et al. analyze plasma amino acid levels in GBM patients versus a control group, suggesting that plasma free amino acid profiling may have prognostic value [86]. In addition, Ferrasi et al. identify seven metabolites as GBM markers, emphasizing their involvement in crucial cellular processes, such as energy metabolism, epigenetic regulation, protein turnover, and signaling pathways that promote cell proliferation and invasion [87].

Another study investigates the role of biogenic amines in cellular metabolism within GBM patients harboring wildtype *IDH*, tracking stages across various treatment stages. This research reveals significant alterations in plasma metabolite levels associated with surgery, radiation, and chemotherapy. Specifically, two days after surgery, 12 metabolites increase and 11 decrease. The combined treatment with chemoradiation results in an elevation of three and a reduction of three metabolites. These findings prompted the authors to characterize distinct plasma biogenic amine signatures throughout the treatment trajectory of GBM patients [88]. The same research group further characterizes the metabolic changes occurring at distinct stages of the standard treatment protocol for GBM patients with wildtype *IDH* by analyzing the plasma metabolome at three time points: pre-surgery, two days post-surgery (before chemoradiation), and immediately following chemoradiotherapy. A comparative analysis reveals a significant elevation in 15 metabolites in post-radiation compared to pre-radiation samples. Furthermore, a notable decrease in 3-aminopiperidine 2,6-dione is observed after chemoradiation. This study effectively delineates distinct blood metabolic signatures across these treatment stages in GBM patients [89].

Other studies have investigated plasma metabolomic profiles in malignant glioma patients, focusing on correlations with *IDH* mutational status. Zhao et al., for example, observe elevated levels of arginine, N-acetylputrescine, nicotinate, glucosamine, methionine, and trimethylamine-N-oxide, in the plasma of patients with *IDH*-mutant gliomas compared to those with wildtype *IDH* [82]. A separate study demonstrates that *IDH* mutation triggers a significant reduction of the activity of pyruvate dehydrogenase (PDH), the enzyme that catalyzes the decarboxylation of pyruvate to acetyl CoA prior to entry into the TCA cycle, thus inducing a metabolic reprogramming of pyruvate which is crucial for cell proliferation and clonogenicity, offering a swift potential for therapeutic intervention [42].

Other investigators have explored the plasma metabolome in 159 GBM patients to identify metabolites associated with two-year OS and progression-free survival (PFS). This investigation demonstrated that elevated kynurenate levels significantly correlated with reduced two-year OS and PFS rates. Conversely, increased levels of arginine and methionine were associated with improved two-year OS and PFS outcome [90]. Bafiti et al. categorized GBM patients into low- and high-risk groups based on their OS exceeding or falling below twelve months, respectively. Their analysis revealed that the low-risk patient cohort exhibited significantly higher levels of 2,6-diisopropyl-3-methylphenol, 7-ketocholesterol, perlolyrine, piperidine, and dopamine. Conversely, the high-risk patient group exhibited elevated levels of (±)-(Z)-2-(5-Tetradecenyl) cyclobutanone and hippuric acid [91].

Furthermore, a pilot study investigates metabolic profiles in GBM patient plasma and saliva samples collected both pre- and post-operatively. Patients were stratified based on PFS into “good” (≥ 9 months) and “poor” (< 9 months) outcome groups. A total of 151 metabolites and 197 lipids were identified in both saliva and plasma samples, with some linked to poor prognosis [29].

While peripheral blood, saliva, and urine are often preferred in metabolomics studies due to ease of collection, CSF, given its direct proximity to CNS, may provide a more accurate representation of the physiological changes occurring within the CNS compared to other biofluids [92]. However, collection of CSF samples presents significant logistical and ethical challenges, particularly in studies involving healthy participants [93].

Metabolomic analysis of CSF from glioma patients reveals: (i) higher levels of citric and isocitric acids in GBM compared to grade I-III gliomas; (ii) increased 2-aminopimelic acid and lactate in GBM versus grade I-II gliomas; and (iii) elevated citric acid, isocitric acid, and lactate in *IDH*-mutant grade I-III gliomas compared to *IDH*-wildtype counterparts [94]. Afterward, another group assessed the CSF metabolome in GBM patients versus a control group undergoing neurosurgery for other entities, as nonglial tumors or hydrocephalus. This analysis reveals distinct metabolic profiles, emphasizing higher levels of glutamate and putrescine as potential diagnostic CSF markers for GBM [95].

Overall, these data from biological fluids highlight specific metabolites correlated to tumor subtypes, therapeutic treatments and patient outcomes, underlining their involvement mainly in the Warburg effect, along with the modulation of the citric acid cycle and the metabolism of amino acids, GSH, glycerophospholipids and pyrimidines.

## Metabolomics evaluations in clinical settings

In clinical settings, metabolomic evaluations is obtained via MRSI, a non-invasive method used to diagnose GBM and generate metabolite maps from a single volume or multiple volume elements across the entire brain [96]. A number of studies have identified metabolites useful for diagnosis and prediction of treatment response and patient outcome (Table 5).

**Table 5:** Summary of the metabolomics studies in clinical setting by MRSI

Comparisons	Metabolite levels	References
**GBM** ***versus*** **healthy tissue voxels**	↑ Choline, and phosphocholine;	[96]
	↓ NAA	
	↑ Choline	[107]
	↑ Glycine, glutamine, taurine, lactate/creatine, phosphocholine/creatine, phosphocholine/NAA, lactate/ NAA, and glutamine/glutamate;	[99]
	↓ Creatine, glutamate, NAA, N-Acetylaspartylglutamic acid, and glycine/taurine	
**Peritumoral area** ***versus*** **metastasis**	↑ Choline	[100]
	↑ Choline/creatine, and choline/NAA;	[101]
	↓ NAA/creatine	
**Pediatric GBM**	↑ Choline, and lactate;	[102]
	↓ NAA	
***IDH*** **mutant** ***versus IDH*** **wildtype**	↑ 2-HG	[105]
**Outcome**		
*Radiotherapy-induced changes versus tumor recurrence*	↓ Choline, and creatine	[107]
*Earlier recurrence and poor prognosis*	Choline/NAA > 1.31	[108]
*Recurrent tumor versus post-treatment radiation effects*	Choline/creatine > 1.54, Creatine/choline ≤ 0.63, lactate/choline ≤ 2.67, lactate/lipids ≤ 1.64, lipids/lactate > 0.58,	[110]
*Relapse versus no relapse*	lactate/NAA ≥ 0.4	[109]
	↑ Choline/NAA	[111]
*Poor versus good survival*	↑ Lactate	[104]

In this context, higher levels of choline and phosphocholine and lower levels of N-Acetylaspartic acid (NAA) within a lesion suggest the presence of high-grade glioma, whereas lower levels of choline and NAA are associated with less metabolic activity (i.e. lower grade) and necrosis [97]. In detail, a very high choline/NAA ratio (generally > 2.2) is particularly indicative of a high-grade neoplasm [98]. However, high levels of choline and low levels of NAA may also represent a cerebral infarct [99].

Lower levels of creatine, glutamate, NAA, N-Acetylaspartylglutamic acid, and glycine/taurine ratio, and higher levels of glycine, glutamine, taurine, lactate/creatine, phosphocholine/creatine, phosphocholine/NAA, lactate/NAA, and glutamine/glutamate ratios are found in GBM voxels compared to healthy tissue voxels [100]. Higher levels of choline were found in the peritumoral area of GBM when compared with brain metastasis [101]. Moreover, lower peritumoral levels of NAA/creatine, and higher of choline/creatine, and choline/NAA can distinguish GBM from intracranial metastases [102]. Marked lower levels of NAA, and higher levels of choline and lactate are found also in pediatric GBM [103]. In detail, choline/creatine ratio > 2.48 is specifically useful in identifying metastases [104].

Considering the compartment characterized by the lowest relative cerebral blood volume and the lowest apparent diffusion coefficient calculated from perfusion and diffusion imaging, some authors demonstrate that higher lactate levels in this compartment are associated with worsened survival in GBM patients [105].

In glioma tissues with *IDH* mutations, higher levels of 2-HG have been quantified. The sensitivity of MRSI to detect 2-HG depends on tumor volume, with lower detection sensitivity for this oncometabolite is found in larger tumors (> 8 ml). Since the levels of 2-HG decrease during cytoreductive therapy, this metabolite represents a valuable biomarker for monitoring treatment response and potential relapses in *IDH*-mutant solid tumors [106]. 2-HG is also shown to be valuable for radiotherapy planning [107].

MRSI can also discriminate between radiotherapy-induced changes (pseudoprogression) and tumor recurrence. Specifically, elevated choline levels indicate tumor progression or recurrence [108], and, in particular, ratio of choline/NAA > 1.31 in the post-operative peritumoral zone predicts earlier recurrence and is associated with poor prognosis [109]. Conversely, lower levels of choline and creatine suggest radiation necrosis or partial remission [108]. Deviers et al. report that a lactate/NAA concentration ratio > = 0.4 in voxels prior to radiotherapy can predict relapse in GBM patients, with higher levels associated with the radioresistant tumor portions [110]. Moreover, the concentration ratio of (choline/creatine) > 1.54, (creatine/choline) ≤ 0.63, (lactate/choline) ≤ 2.67, (lactate/lipids) ≤ 1.64 and (lipids/lactate) > 0.58 can distinguish recurrent tumor from post-treatment radiation effect groups [111]. Other authors, after analyzing the sites of relapse from GBM patients treated with Tipifarnib and radiotherapy, showed that metabolically active regions with high choline/NAA ratio are predictive of post- radiotherapy relapse sites [112].

Overall, these data highlight a key role of some metabolites like NAA, choline, creatine and lactate as diagnostic and prognostic markers for GBM and confirm the correlation between higher levels of 2-HG and the presence of *IDH* mutation.

## Discussion

**Fig. 2:**
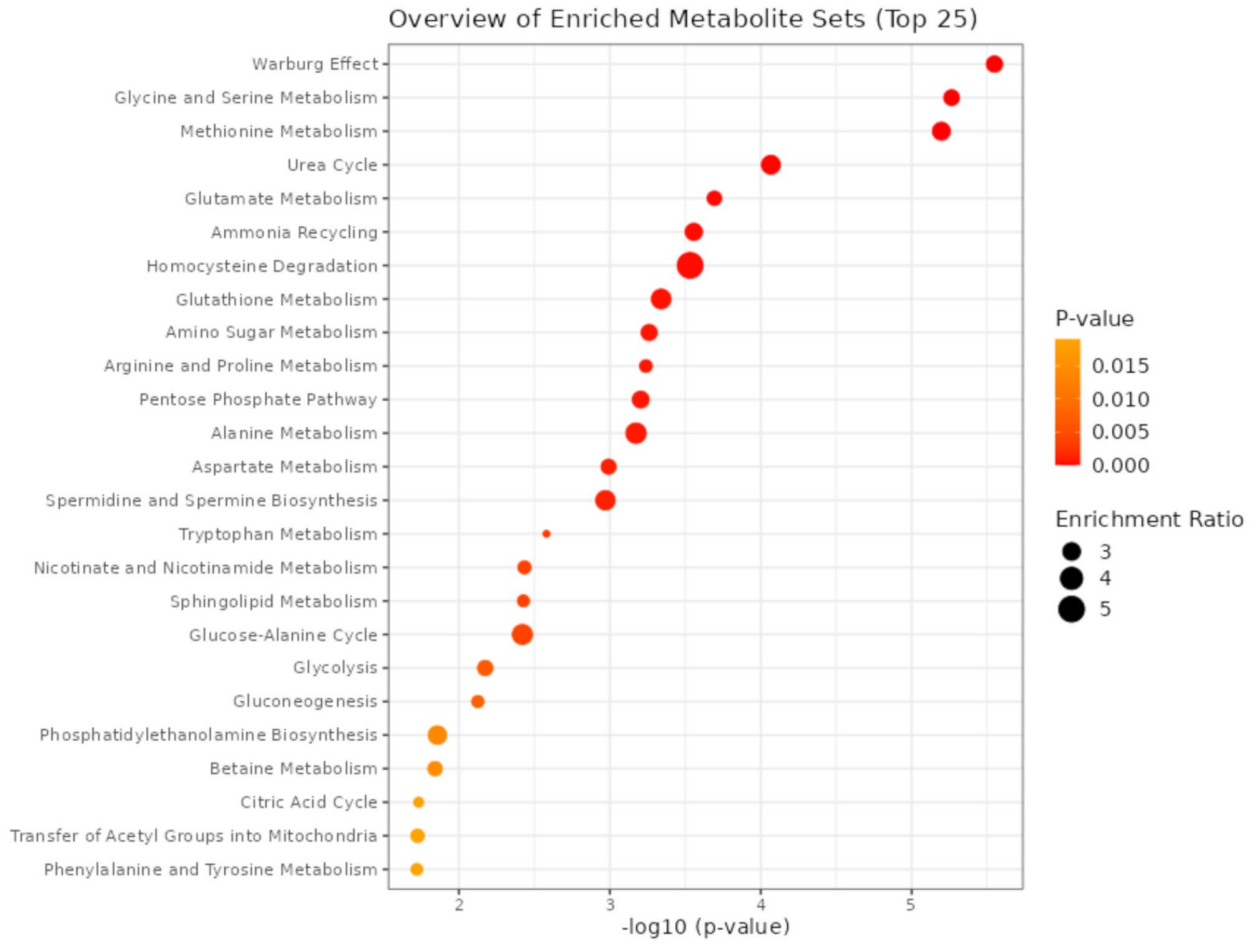
Enriched pathway analysis by dot plot. Colors, from yellow to red, and increasing node size indicate increasing enrichment of the pathways with *p* values < 0.05

To get an integrate picture of the metabolomic alterations associated with malignant gliomas and GBM, we performed a pathway analysis that highlights the biological functions overrepresented in the group of all dysregulated metabolites, ranking these functions by the relative abundance of metabolites pertinent to each specific pathway using statistical methods (Fig. 2). It appears that the metabolomic reprogramming of cancer cells primarly centers on the Warburg effect, an enhancement of glycolysis and lactic acid production associated with decreased oxidative phosphorylation and TCA cycle activity also in the presence of oxygen availability and a functional mitochondrial apparatus. Such a peculiar behavior is driven by the need for huge amounts of building blocks (e.g., nucleic acids, lipids, amino acids) by rapidly proliferating cancer cells. The Warburg effect is known to promote tumor progression by enhancing angiogenesis, tumor-associated fibroblast formation, immunosuppression, and drug resistance; undeniably, the chance to target this metabolic reprogramming holds significant therapeutic potential [113]. The ketogenic diet, which elevates ketone bodies production and lowers glucose availability, insulin levels, oxidative stress, inflammation and proteins related to angiogenesis, offers a potential additional, non-pharmacological approach for managing malignant glioma and GBM through modulation of the Warburg effect [114]. Pharmacological interventions targeting the Warburg effect have also demonstrated efficacy; indeed, Aurora kinase A genetic and pharmacological inhibition reduces glycolysis and triggers metabolic reprogramming by activating Peroxisome Proliferator-Activated Receptor (PPAR)α and inhibiting MYC targets [115].

The Warburg effect is intricately linked to several other metabolic pathways and contributes significantly to GBM aggressiveness by its intricate linkage with alternative metabolic pathways, notably the PPP. Increased glycolysis in GBM cells provides substrate for the PPP where, under normal oxygen conditions, exhibit elevated PPP enzymes expression. Conversely, in a hypoxic environment, a metabolic shift towards increased glycolysis and relatively decreased PPP activity occurs, as an adaptation facilitating cell migration and invasion, thereby promoting GBM progression [116–118]. Indeed, despite its anti-angiogenic effects, bevacizumab treatment in recurrent GBM has been observed to worsen patient prognosis [119], reasonably through the hypoxia-induced subsequent lactate accumulation, which can favor highly glycolytic, highly malignant cellular clones and also generate an immunosuppressive microenvironment.

Additionally, glutamate metabolism plays a crucial role in GBM, where the Warburg effect supports oncogenesis also through *glutamic pyruvate transaminase 2* (*GPT2*)-mediated coupling of pyruvate production to glutamine catabolism [120]. Glutamate, a nitrogen transporter and the brain’s main excitatory neurotransmitter, damages tissue and contributes to the onset of epilepsy, a frequent clinical manifestation in malignant glioma patients [120]. This metabolite is also critical for the neuron-GBM cell cross-talk [120]. Indeed, several authors demonstrate that the neurons form glutamatergic synaptic connections onto oligodendrocyte precursor cell (OPC) proliferation [121], and that synaptic transmission occurs between glutamatergic neurons and a subset of xenografted human glioma cells, exhibiting properties similar to synapses formed with normal OPCs [122]. Evidence suggests a role for glutamate receptors in the biology of cancer and glioma, and reveals that all glutamate receptor subunits are differentially expressed in the tumor cell lines. Their expression is associated with the formation of functional channels suggesting their antagonists can represent a feasible goal to be explored in clinical trials [123]. However, pharmacological targeting of glutamate metabolism is therapeutically challenging, due to the pronounced side effects of the anti-glutamatergic drugs [124] and the complex glioma microenvironment [125]. Glutamate metabolism is further connected with GSH metabolism. High GSH levels, often observed in the hypoxic GBM environment, reduce the effectiveness of many anti-tumor drugs [126]. Key players in GSH metabolism also include γ-glutamyl transferase, overexpressed in GBM, which maintains GSH homeostasis and protects tumor cells from oxidative stress [127]. Similarly, the cystine/glutamate transporter xCT (SLC7A11), highly expressed in GBM, imports cystine for GSH synthesis, bolstering tumor cells resistance to oxidative damage [128]. Activation of the N-metil-D-aspartate receptor (NMDAR) glutamate receptor, further enhances antioxidant capacity by upregulating the GSH redox cycle, thus contributing to tumor progression and therapy resistance [129, 130]. Additionally, the activity of the α-amino-3-hydroxy-5-methyl-4-isoxazolepropionic acid receptor (AMPAR) glutamate receptor, is strictly correlated with brain tumor progression, and AMPAR inhibitor perampanel reduces glioma cell growth and invasive behavior [131].

Furthermore, glutamate metabolism is linked to ammonia recycling. Glutamine synthase (GS), which normally recycles ammonia and glutamate into glutamine, is upregulated in GBM, with lower GS levels associated with longer GBM patient survival [132]. Conversely, the urea cycle enzyme, Carbamoyl-Phosphate Synthase (CPS)1, that transforms ammonia and bicarbonate into carbamoyl phosphate, is elevated in GBM promoting cell proliferation, migration, and invasion [133]. High urea levels correlate with tumor markers, as well as Ki67 and *MGMT*, supporting the rationale of investigating urea-lowering drugs in combination with chemotherapy [134].

Several other amino acid metabolic pathways are dysregulated in GBM, contributing to tumor progression and immune evasion [135]. Glycine and serine metabolism are frequently upregulated, especially in tumors with *EGFR* and *Phosphoserine Phosphatase* (*PSPH)* mutations, sustaining tumor growth, migration, and immune evasion [136, 137]. GBM growth is also dependent on methionine, with tumor aggressiveness linked to this dependence and the folate cycle. Indeed, folate supplementation restores GBM stem cell growth and promotes tumor sphere formation, raising concerns about its potential role in disease progression. This suggests a straightforward link with vitamin supplements as well as an additional connection between dietary intake and tumor development [138–140].

Altered arginine metabolism, characterized by increased arginine transporter expression and arginase activity, promotes immunosuppression and tumor growth [141]. Similarly, disrupted proline and alanine metabolism contribute to tumor growth, aggressiveness, and inflammation, suggesting a key role for these pathways in malignant glioma progression [142–144].

Finally, tryptophan metabolism plays a key role in establishing an immunosuppressive tumor microenvironment. This occurs not only through indoleamine 2,3-dioxygenase 1 (IDO1) -mediated degradation, but also via the production of kynurenine and kynurenic acid, which activate the aryl hydrocarbon receptor [145]. Given the compartmentalization of tryptophan metabolism in the CNS and its impact on brain function [146], targeting this pathway is of undoubted therapeutic interest. Indeed, IDO1 inhibitors are currently in clinical trials for GBM [147, 148] and the kynurenine/tryptophan ratio is a promising prognostic and predictive biomarker in GBM patients treated with surgery and immunotherapy [149].

A crucial point for discussion is the role of 2-HG in *IDH* mutated gliomas; indeed its higher levels are consistently reported across in vitro [42] and in vivo [63] models, tumor tissues [72, 75, 76], and plasma [82]. 2-HG has a structure similar to α-ketoglutarate, which is an intermediate product of TCA cycle, produced by the oxidative decarboxylation of isocitrate: this reaction is catalyzed by IDH. It has been reported that *IDH* mutation lead to the production of a mutant enzyme with a novel neomorphic activity; in particular, the mutant enzyme catalyzes the reduction of α-ketoglutarate to an abnormal metabolite known as 2-HG which accumulates in tumor cells and plays a key role in driving tumorigenesis [150]. Indeed, the significant role of 2-HG in cancer was demonstrated for first time in 2-hydroxyglutaric aciduria, tumors harboring *IDH* mutations [151]. 2-HG has been verified to accumulate in the context of hypoxia or acidic pH, and play a crucial role in the fate decision of immune cells [151]. Higher levels of 2-HG have effects on GBM biology by acting as a competitive inhibitor of α-ketoglutarate–dependent dioxygenases. This inhibition leads to widespread epigenetic dysregulation, including histone and DNA hypermethylation, which alters gene expression patterns and promotes a block in cellular differentiation [152]. Overall, *IDH* mutations together with higher levels of 2-HG contribute to gliomagenesis by inducing epigenetic reprogramming, impairing cell differentiation, and altering the tumor microenvironment, and are potential interesting targets for diagnostic and therapeutic strategies in glioma treatment.

Collectively, all these findings highlight how identifying changes in key metabolites, including lipids, nucleotides, carbohydrates and amino acids, is valuable for understanding the mechanisms underlying metabolic reprogramming in GBM. This understanding, in turn, paves the way for advanced clinical strategies targeting multiple interconnected pathways.

## Conclusions

This review summarizes current metabolomics methodologies and research in GBM, examining diverse biological matrices, such as in vitro and in vivo models, tissues, and biofluids. These analyses consistently reveal metabolic variations that not only distinguish GBM samples from normal brain tissues but also correlate with specific aggressive factors like *MGMT* promoter methylation, *IDH* mutation status and stemness. All metabolomics approaches converge in identifying key metabolite variations regardless of biological sample type, thereby enabling the discovery of crucial prognostic, diagnostic, predictive, and therapeutic biomarkers. The present data collectively indicate that metabolic reprogramming in GBM cells is characterized by dysregulation in multiple pathways, particularly glycolysis (Warburg effect), amino acid metabolism, and the urea cycle. Ultimately, these identified metabolic changes disclose promising tumor targets - lipids, nucleotides, carbohydrates, and amino acids– thus driving the development of novel therapeutic strategies.

## Data Availability

No datasets were generated or analysed during the current study.

## Abbreviations

2D: Two-dimensional
2-HG: 2-hydroxyglutarate
3D: Three-dimensional
AEG-1: Astrocyte elevated gene-1
AMP: Adenosine MonoPhosphate
AMPAR: α-amino-3-hydroxy-5-methyl-4-isoxazolepropionic acid receptor
ATP: Adenosine triphosphate
BBB: Blood–brain barrier
BT-RADS: Brain tumor - reporting and data system
Cas CRISPR: Associated system
CDKN2A/2B: Cyclin-dependent kinase inhibitor 2 A/2B
CNS: Central nervous system
CPS1: Carbamoyl-Phosphate Synthase 1
CRISPR: Clustered Regularly Interspaced Short Palindromic Repeats
CSC: Cancer stem cell
CSF: Cerebrospinal fluid
CTLA-4: Cytotoxic T-Lymphocyte Antigen 4
DHAP: Dihydroxyacetone phosphate
EGFR: Epidermal growth factor receptor
EGFR: Epidermal Growth Factor Receptor
G3P: Glycerol-3-phosphate
G6P: Glucose-6-phosphate
GBM: Glioblastoma
GC: Gas chromatography
GPT2: Glutamic pyruvate transaminase 2
GS: Glutamine synthase
GSCs: Glioma stem cells
GSH: Glutathione
GSSG: Glutathione disulfide
HPLC: High-Performance LC
HRMAS: High resolution magic angle spinning
ICI: Immune checkpoint inhibitor
IDH: Isocitrate dehydrogenase
IDO1: Indoleamine 2,3-dioxygenase 1
IRF9: Interferon regulatory factor 9
Kras: Kirsten rat sarcoma virus
LC: Liquid chromatography
MGMT: O6-methylguanine-DNA methyltransferase
miRNA: Micro RNA
MRI: Magnetic resonance imaging
MRSI: Magnetic resonance spectroscopy imaging
MS: Mass spectrometry
MSC: Mesenchymal stem cells
NADP: Nicotinamide Adenine Dinucleotide
NANO: Neurologic assessment in neuro-oncology
NMDAR: N-metil-D-aspartate receptor
NMR: Nuclear magnetic resonance
OPC: Oligodendrocyte Precursor Cell
OS: Overall survival
PAM: Plasma-activated medium
PD-1: Programmed cell death protein 1
PDGFRA: Platelet-Derived Growth Factor Receptor alpha
PDH: Pyruvate dehydrogenase
PFS: Progression-free survival
PPAR: Peroxisome Proliferator-Activated Receptor
PPP: Pentose phosphate pathway
PSPH: Phosphoserine Phosphatase
PTEN: Phosphatase and tensin homolog
SLC1A: Solute Carrier Family 1 Member 1
SLC7A8: Solute Carrier Family 7 member 8
SLC7A11: Cystine/glutamate transporter xCT
SLC38A1: Solute Carrier Family 38 member 1
TCA: Tricarboxylic acid cycle
TERT: Telomerase reverse transcriptase
TMZ: Temozolomide
TOF: Time-of-flight
UDP: Uridine diphosphate
UPLC: Ultra-Performance liquid chromatography
VEGF: Vascular Endothelial Growth Factor
XRCC1: X-ray repair cross-complementing 1
